# Airway microbiota in young people across four continents differ by country, asthma status and inflammatory phenotype

**DOI:** 10.1136/thorax-2025-222965

**Published:** 2026-01-30

**Authors:** Steven L Taylor, Collin R Brooks, Lucy Pembrey, Sarah K Manning, Levi Elms, Harriet Mpairwe, Camila A Figueiredo, Aida Y Oviedo, Martha Chico, Jeroen Burmanje, Hajar Ali, Irene Nambuya, Pius Tumwesige, Steven Robertson, Charlotte E Rutter, Karin van Veldhoven, Susan M Ring, Mauricio L Barreto, Philip J Cooper, Álvaro A Cruz, Neil Pearce, Geraint B Rogers, Jeroen Douwes, the WASP Study Group, Neil Pearce

**Affiliations:** 1 Microbiome and Host Health Programme, South Australian Health and Medical Research Institute, Adelaide, South Australia, Australia; 2 College of Medicine and Public Health, Flinders University, Adelaide, South Australia, Australia; 3 Research Centre for Hauora and Health, Massey University, Wellington, New Zealand; 4 London School of Hygiene & Tropical Medicine, London, UK; 5 MRC/UVRI and LSHTM Uganda Research Unit, Entebbe, Uganda; 6 Federal University of Bahia Salvador, Salud, Brazil; 7 Fundación Ecuatoriana Para Investigación en Salud, Quito, Ecuador; 8 Population Health Sciences, Bristol Medical School, University of Bristol, Bristol, Bristol, UK; 9 MRC Integrative Epidemiology Unit, University of Bristol, Bristol, UK; 10 Center for Data and Knowledge Integration for Health, Fiocruz, Bahia, Bahia, Brazil; 11 Institute of Infection and Immunity, St George’s University of London, London, UK; 12 School of Medicine, Universidad Internacional del Ecuador, Quito, Pichincha, Ecuador; 13 Medicine, Federal University of Bahia, Salvador, Bahia, Brazil

**Keywords:** Microbiota, Asthma, Asthma Epidemiology, Asthma Mechanisms

## Abstract

**Background:**

Asthma is an umbrella diagnosis encompassing distinct pathophysiological mechanisms. While a global problem, our understanding of the interplay between respiratory microbiology and airway inflammation is largely from populations in high-income settings. As a result, treatment approaches align poorly with asthma characteristics in less studied populations.

**Objective:**

To identify conserved and geographically distinct relationships between airway inflammation and microbiota characteristics in young people with and without asthma.

**Methods:**

We conducted a cross-sectional study performing inflammatory phenotyping, microbiota analysis and enumeration of total bacteria, *Haemophilus influenzae* and *Moraxella catarrhalis* on 488 induced sputum samples from participants from Brazil (asthma: 68; non-asthma: 8), Ecuador (asthma: 89; non-asthma: 30), Uganda (asthma: 61; non-asthma: 8), New Zealand (asthma: 129; non-asthma: 58) and the UK (asthma: 25; non-asthma: 20). Microbiota characteristics were compared by country, asthma status and inflammatory characteristics, adjusting for age and sex.

**Results:**

Asthma inflammatory phenotypes and microbiology differed between countries, with Uganda characterised by higher neutrophils, microbial diversity and bacterial abundance. Comparison of airway inflammation with microbiota characteristics showed conserved relationships across centres, with airway neutrophil proportion explaining variance in microbiota Bray-Curtis dissimilarity (p<0.001) and being positively associated with bacterial abundance, including *H. influenzae* and *M. catarrhalis* load (all p<0.05). In contrast, eosinophil proportion was less strongly associated with microbiota dissimilarity (p=0.033) and only associated with *Streptococcus* abundance. Country-specific associations between airway inflammation and microbiology were evident.

**Conclusion:**

Both airway inflammation and microbiology varied geographically in young people with asthma. Associations between microbiota characteristics and neutrophilic phenotype were conserved.

**WHAT IS ALREADY KNOWN ON THIS TOPIC**

- Asthma treatment response and severity are associated with airway inflammation and microbiology.
- Most asthma research is performed in high-income countries, and the generalisability in other settings is unclear.

**WHAT THIS STUDY ADDS**

- Asthma inflammatory phenotypes and airway microbiota vary across high income (New Zealand and the UK) and low to middle income (Brazil, Ecuador and Uganda) countries.
- The association between airway microbiota and neutrophilic and eosinophilic inflammation is complex and varied between countries.

**HOW THIS STUDY MIGHT AFFECT RESEARCH, PRACTICE OR POLICY**

- Understanding variation in underlying pathophysiology between countries can inform improved deployment of maintenance asthma therapies, such as macrolides and inhaled corticosteroids, that target specific inflammatory pathways.

## Introduction

 Asthma is associated with several distinct pathophysiological presentations (deemed inflammatory phenotypes), including type 2 (T2)-high/eosinophilic and neutrophilic disease. Different inflammatory phenotypes are associated with variations in treatment response and provide a basis for targeted treatment strategies. For example, inhaled corticosteroid (ICS) therapy is typically effective for those with early-onset, eosinophilic asthma (EA), but provides limited benefit in those with late-onset EA, non-T2-high (T2-low) or neutrophilic asthma (NA).^1^

Airway microbiology varies with asthma inflammatory phenotypes,^2–5^ likely reflecting differences in airway pathophysiology and underlying aetiology. For example, lower microbiota diversity and increased prevalence of opportunistic pathogens, including *Haemophilus influenzae* and *Moraxella catarrhalis*, have been associated with NA.^2–6^ Airway microbiology has been associated with differential response to macrolide maintenance therapy in adults with poorly controlled asthma,^7^ outperforming endotype classification or inflammatory cell-based prediction of treatment response.^8^

While the distribution of asthma subtypes is now well-documented within some populations, particularly those in high-income countries (HICs),^9–12^ far less is known about asthma characteristics in low and middle-income countries (LMICs). Recently, we reported considerable variance in asthma phenotypes between Brazil, Ecuador, New Zealand, Uganda and the UK,^9^ suggesting that factors, such as environmental exposures or socioeconomic status, may influence patterns of asthma pathophysiology. Although no other study has examined asthma phenotype prevalence comparing HICs and LMICs on the basis of airway inflammation, our observations are consistent with the earlier findings of the International Study of Asthma and Allergic Diseases in Childhood (ISAAC). Specifically, ISAAC reported that the proportion of asthma attributable to atopy differs between countries, with an average population attributable fraction of 46% observed in HICs and 20% in LMICs.^13^

The extent to which geographic variation in asthma phenotype prevalence is reflected in differences in airway microbiology, and how any such differences relate to treatment outcomes and asthma-associated complications, remains unclear. In this component of the World Asthma Phenotypes (WASP) study, termed WASP-biome, we assessed the airway microbiota characteristics of children and young adults with and without asthma in Brazil, Ecuador, New Zealand (NZ), Uganda and the UK. Our principal aim was to identify conserved and geographically distinct relationships between inflammatory phenotypes and airway microbiota characteristics in young people with asthma.

## Methods

Detailed methods are provided in the online supplement.

### Study design

The WASP study is a cross-sectional observational investigation of asthma phenotypes in young people in HICs and LMICs.^14^ The study was conducted in five centres: Bristol, UK (Avon Longitudinal Study of Parents and Children)^15–17^; Wellington, New Zealand; Salvador, Brazil; Quinindé, Ecuador and Entebbe, Uganda (table 1).^9^ WASP-biome involves a subgroup of participants for whom a secondary sputum aliquot was available for microbiota analysis. No other selection criteria were defined for inclusion in WASP-biome.

**Table 1:** Study characteristics of young people with asthma

	Brazil (Salvador)	Ecuador (Quininde)	New Zealand (Wellington)	Uganda (Entebbe)	UK (Bristol)
N	60	89	129	61	25
Female, n (%)	40 (67.0%)	41 (46.0%)	55 (42.6%)	45 (73.8%)	17 (68.0%)
Age (years), median (IQR)	17.98 (16.4–19.6)	11.58 (10.79–13.65)	11.49 (9.97–14.99)	15.09 (14.01–16.51)	26.00 (25.58–26.25)
BMI (kg/m^2^), median (IQR)	22.63 (20.5–25.4)	18.76 (17.33–21.94)	19.39 (17.08–22.48)	20.18 (18.26–22.98)	26.53 (22.68–31.25)
ACQ7 score, median (IQR)	0.67 (0.17–1.50)	0.00 (0.00–0.00)	0.86 (0.33–1.29)	0.83 (0.00–1.67)	0.67 (0.17–1.17)
ACQ7 level, n (%)					
Well controlled	43 (72.9%)	84 (94.4%)	100 (82.0%)	41 (67.2%)	22 (88.0%)
Uncontrolled	16 (27.1%)	5 (5.6%)	22 (18.0%)	20 (32.8%)	3 (12.0%)
Unknown	1 (1.7%)	0 (0%)	7 (5.4%)	0 (0%)	0 (0%)
ICS use past 12 months, n (%)					
Yes	10 (16.7%)	6 (6.7%)	84 (65.1%)	3 (4.9%)	15 (60.0%)
No	48 (80.0%)	50 (56.2%)	44 (34.1%)	55 (90.2%)	5 (20.0%)
Unknown	2 (3.3%)	33 (37.1%)	1 (0.8%)	3 (4.9%)	5 (20.0%)
SABA use past 12 months, n (%)					
Yes	31 (51.7%)	13 (14.6%)	118 (91.5%)	19 (31.1%)	21 (84.0%)
No	27 (45%)	43 (48.3%)	10 (7.8%)	41 (67.2%)	0 (0%)
Unknown	2 (3.3%)	33 (37.1%)	1 (0.8%)	1 (1.6%)	4 (16.0%)
FEV_1_ (% predicted), mean (SD)†	91.47 (10.10)	98.04 (11.52)	92.93 (13.10)	97.67 (11.05)	97.87 (13.80)
FVC (% predicted), mean (SD)†	96.72 (15.08)	96.36 (10.77)	99.97 (10.94)	101.91 (12.67)	103.69 (13.75)
Atopy, n (%)					
Yes	51 (85.0%)	29 (32.6%)	99 (76.7%)	31 (51.7%)	15 (78.9%)
No	9 (15.0%)	60 (67.4%)	30 (23.3%)	29 (47.5%)	4 (16.0%)
Unknown	0 (0%)	0 (0%)	0 (0%)	1 (1.6%)	6 (24.0%)
Inflammatory phenotype, n (%)					
Eosinophilic	19 (31.7%)	28 (31.5%)	64 (49.6%)	12 (19.7%)	8 (32.0%)
Neutrophilic	3 (5.0%)	4 (4.5%)	9 (7.0%)	24 (39.3%)	3 (12.0%)
Paucigranulocytic	37 (61.7%)	52 (58.4%)	51 (39.5%)	19 (31.1%)	13 (52.0%)
Mixed granulocytic	1 (1.7%)	5 (5.6%)	5 (3.9%)	6 (9.8%)	1 (4.0%)
Sputum neutrophils (%), median (IQR)	5.75 (1.38–15.62)	10.32 (5.00–22.25)	20.00 (8.48–44.15)	60.59 (30.35–85.00)	28.50 (15.29–46.88)
Sputum eosinophils (%), median (IQR)	1.00 (0.00–4.75)	0.73 (0.12–4.25)	3.25 (0.75–9.25)	0.75 (0.0–3.50)	1.39 (0.25–3.25)

* Missing data from Brazil (n=2), Ecuador (n=1).

† Missing data from Brazil (n=2), Ecuador (n=1), Uganda (n=11).

ACQ7, Asthma Control Questionnaire; BMI, body mass index; FEV1, forced expiratory volume in 1 second; FVC, forced vital capacity; ICS, inhaled corticosteroids; SABA, short acting beta-agonist.

This study was approved by the LSHTM ethics committee (ref: 9776). Informed consent was obtained from all participants or their parents/carers before taking part.

### Asthma diagnosis, sputum collection and inflammatory phenotyping

Asthma was defined across all centres as wheeze or whistling in the chest and/or use of asthma medication in the past 12 months, using the validated ISAAC questionnaire.^18–20^ Induced sputum samples were collected using a standardised protocol.^21^ Stored, unprocessed induced sputum aliquots were used for microbiota analysis. Inflammatory phenotypes were determined from differential cell counts, performed on freshly collected samples. Samples with ≥30% squamous cells were excluded due to suspected high upper airway contamination. Asthma inflammatory phenotypes were defined as: EA: ≥2.5% eosinophils and <61% neutrophils; mixed granulocytic asthma (MGA): ≥2.5% eosinophils and ≥61% neutrophils; NA: <2.5% eosinophils and ≥61% neutrophils; and paucigranulocytic asthma (PGA): <2.5% eosinophils and <61% neutrophils.^9^

### Microbiota characterisation

Induced sputa underwent DNA extraction, quantitative PCR enumeration of total bacteria, *H. influenzae* and *M.catarrhalis* (online supplemental table 1) and 16S rRNA sequencing, all within a single facility. Mock bacterial community (positive control) samples and blank extraction controls were analysed to assess taxon representation and reagent contaminants, respectively. Reads have been deposited in the European Bioinformatics Institute European Nucleotide Archive (PRJEB77703).

### Data analysis

Analyses were conducted to assess microbiota differences (ie, β-diversity, α-diversity, bacterial load and relative abundance of specific taxa) between: (1) different countries (for participants with asthma and without, separately); (2) asthmatics and non-asthmatics (study-wide and within country); (3) asthma inflammatory phenotypes (study-wide and within country) and (4) asthma inflammatory cell percentage (study-wide and within country). All analyses were adjusted for age and sex, with analyses involving multiple countries also adjusted for location. Analysis of airway inflammation was performed based on comparisons between the four inflammatory phenotypes and with percentage neutrophils (neutrophil%) and eosinophils (eosinophil%), mutually adjusted for each other. Benjamini-Hochberg false discovery rate (FDR) correction was applied.

## Results

### Participant characteristics

**Figure 1:**
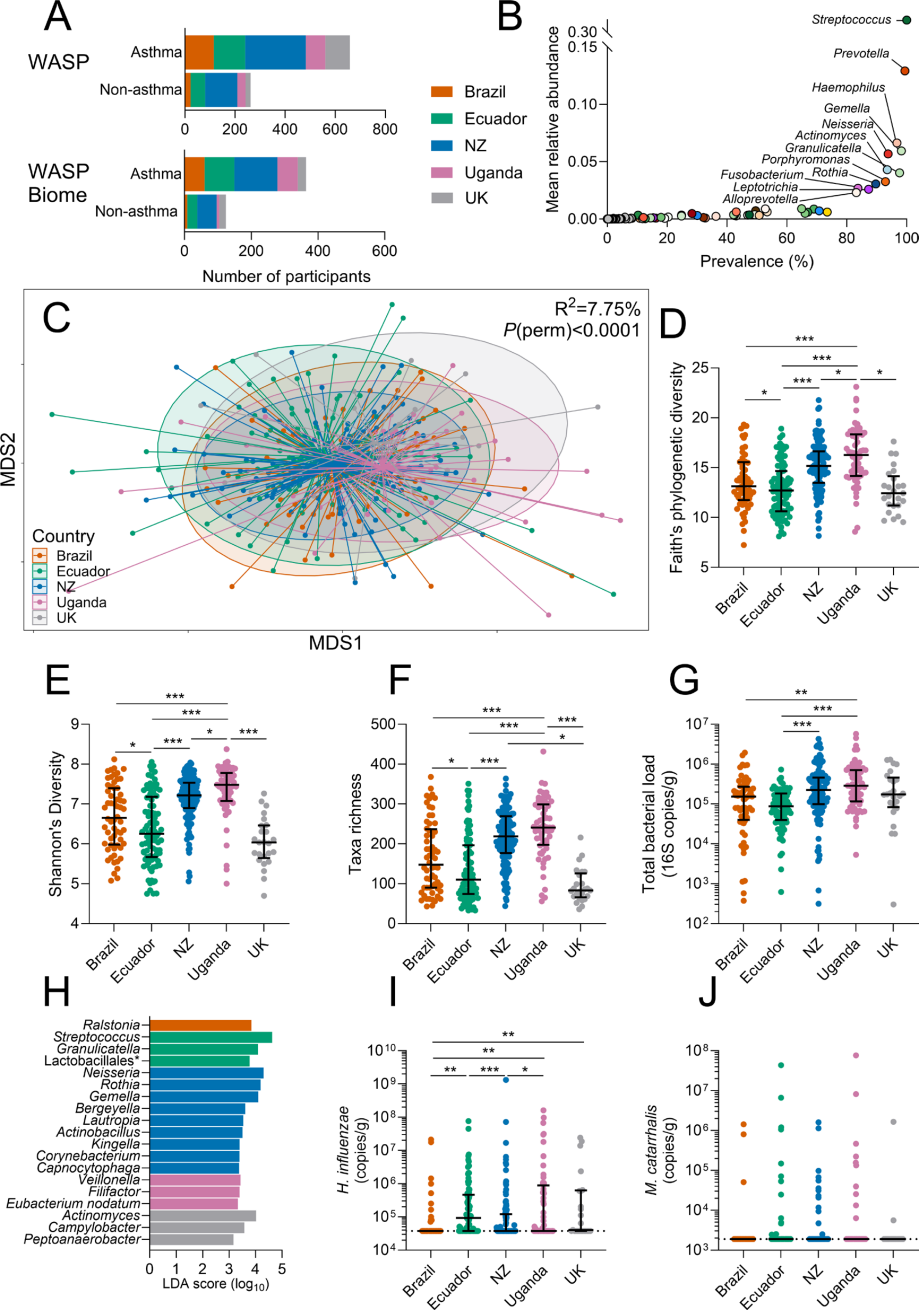
Sputum microbiota differs by country in participants with asthma. (**A**) Overview of sample size for the WASP study and WASP-biome study. (**B**) Taxa summary plot showing the mean relative abundance and prevalence of genera, coloured by phyla (Actinobacteriota: blues, Bacteroidota: oranges, Bacillota: greens, Pseudomonadota: reds, Fusobacteriota: purples, Campilobacterota: yellow, Spirochaetes: brown). (**C**) Non-metric multidimensional scaling (NMDS) plot of Bray-Curtis dissimilarity. (**D**) Faith’s phylogenetic diversity. (**E**) Shannon’s diversity. (**F**) Taxa richness. (**G**) Total bacterial load derived from qPCR. (**H**) Taxa differentially present by country. (**I**) *Haemophilus influenzae* abundance derived from qPCR. (**J**) *Moraxella catarrhalis* abundance derived from qPCR. Statistics: (C) permutational multivariate analysis of variance including variables: country, age, sex; **(D–G, I, J**) ordinal logistic regression including variables: country, age, sex; (**H**) linear discriminant analysis (LDA) effect size, with cut-offs of LDA≥3 and p<0.05. Country colours: orange=Brazil; green=Ecuador; blue=New Zealand; pink=Uganda and grey=UK. *** adjusted p<0.001, ** adjusted p<0.01, * adjusted p<0.05. qPCR, quantitative PCR; WASP, World Asthma Phenotypes.

Of 920 WASP participants, sputum was collected from 658 with asthma and 262 with no asthma. Of these, 488 (asthma n=364, no asthma n=124) provided sufficient, quality sputum for microbiota analysis (WASP-biome). Study participation by centre is shown in figure 1A and WASP-biome participant characteristics are detailed in table 1. WASP-biome subgroup characteristics were consistent with the wider WASP study cohort (online supplemental table 2), with the highest proportion of NA observed in Uganda and the highest proportion of EA in NZ.

### Sputum microbiota characteristics differ between countries

A total of 135 bacterial taxa were identified in participants with asthma. Taxa prevalence and mean relative abundance are shown in figure 1B, and variance between centres is shown in online supplemental figure 1). 46 bacterial taxa were detected in ≥10% of the samples within any of the five centres (online supplemental figure 1). *Streptococcus* was the most commonly detected genus and was present in all samples (mean relative abundance=0.32, SD=0.11), followed by *Prevotella* (prevalence=99.4%), *Gemella* (98.2%)*, Granulicatella* (97.6%)*, Haemophilus* (96.8%)*, Neisseria* (93.8%), *Actinomyces* (93.5%) and *Porphyromonas* (92.9%).

Between-country analysis revealed significant differences in microbiota composition, as defined by Bray-Curtis dissimilarity (R^2^=7.75%, p(perm)<0.0001, figure 1C and online supplemental table 3). Pairwise comparisons showed the greatest difference between NZ and Uganda (pseudo-F=11.5, online supplemental table 3) and the smallest between Brazil and Ecuador (pseudo-F=2.81). Countries also differed in α-diversity indices (Faith’s phylogenetic diversity, Shannon diversity and taxonomic richness count) (figure 1D–F) and total bacterial load (figure 1G), with Uganda having both higher α-diversity and bacterial load, while Ecuador and the UK displaying the lowest. 19 bacterial genera were significantly different between countries (figure 1H), with *Ralstonia*, *Streptococcus*, *Neisseria*, *Veillonella* and *Actinomyces* the highest in Brazil, Ecuador, NZ, Uganda and the UK, respectively. Assessment of the absolute abundance of the Gram-negative respiratory pathogens, *H. influenzae* (figure 1I) and *M. catarrhalis* (figure 1J), revealed *H. influenzae* to be more abundant in participants with asthma in Uganda and Ecuador, compared with Brazil or NZ.

When repeated for non-asthmatic participants, between-country differences were again identified despite the smaller sample size (n=124), particularly for Brazil (n=8) and Uganda (n=8) (online supplemental figure 2A). These differences were most pronounced between Ecuador and NZ (pseudo-F=5.82, p(perm)<0.001) and least evident between Brazil and Uganda (pseudo-F=1.39, p(perm)=0.14, online supplemental table 3). The patterns of α-diversity and total bacterial load in non-asthmatics replicated those with asthma, with Uganda again having the highest α-diversity and bacterial abundance (online supplemental figure B–G).

### Sputum microbiota characteristics differ by asthma status

**Figure 2:**
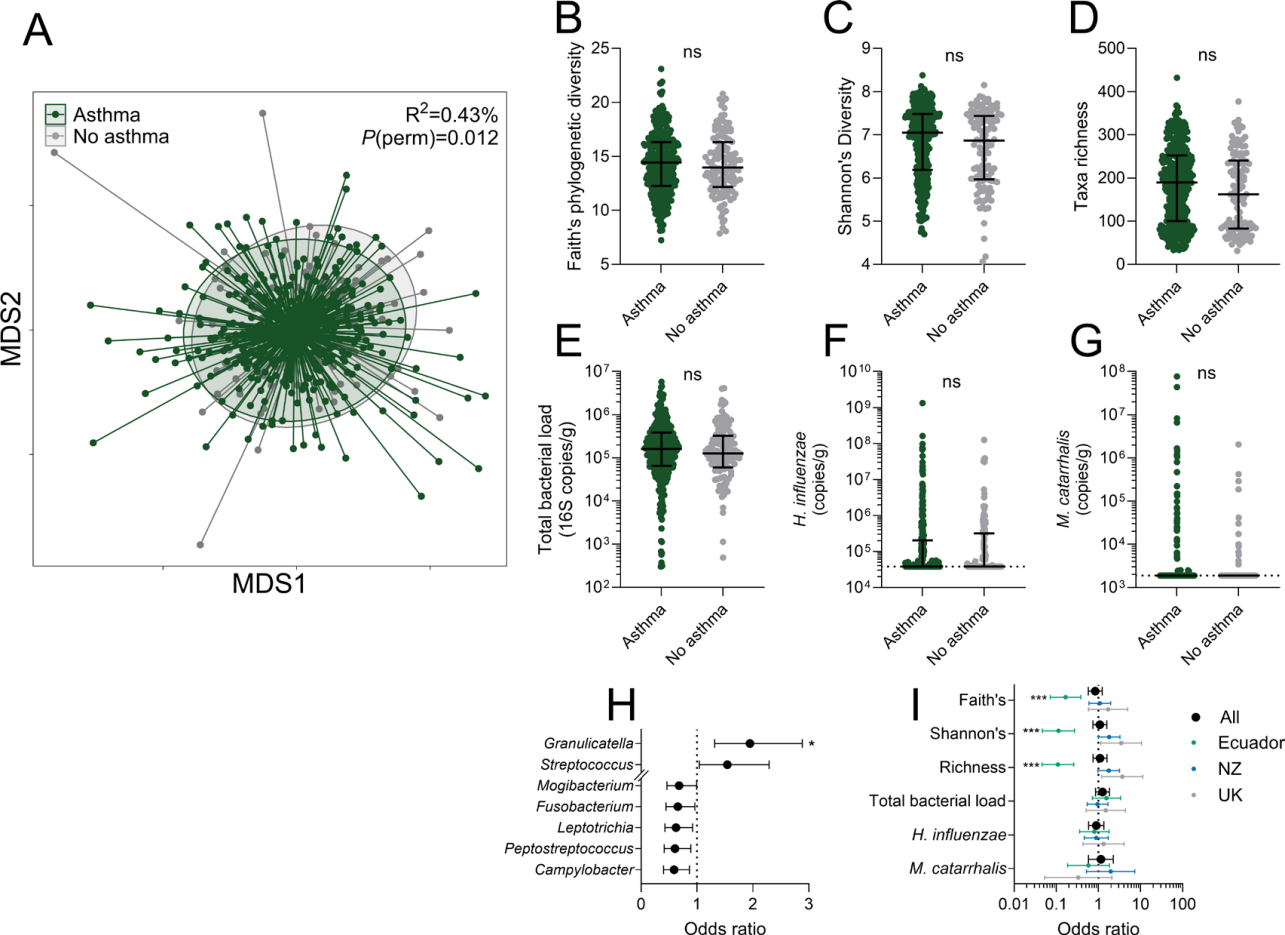
Sputum microbiota differs by asthma status. (**A**) Non-metric multidimensional scaling (NMDS) plot of Bray-Curtis dissimilarity. (**B**) Faith’s phylogenetic diversity. (**C**) Shannon’s diversity. (**D**) Taxa richness. (**E**) Total bacterial load derived from qPCR. (**F**) *Haemophilus influenzae* abundance derived from qPCR. (**G**) *Moraxella catarrhalis* abundance derived from qPCR. (**H**) Forest plot showing taxa that differed significantly by asthma status; left=lower in asthma. (**I**) Forest plot showing association between asthma and α-diversity (Faith’s, Shannon’s, taxa richness) and qPCR derived bacterial load (total, *H. influenzae* and *M. catarrhalis*); left=lower in asthma. Statistics: (A) permutational multivariate analysis of variance including variables: asthma, country, age and sex; (**B–I**) ordinal logistic regression including variables: asthma, country, age and sex; *** adjusted p<0.001, * adjusted p<0.05. qPCR, quantitative PCR.

Microbiota composition differed significantly between participants with asthma and without (R^2^=0.43%, *P* (perm)=0.012, figure 2A and online supplemental table 4). Investigation of compositional differences identified no significant association between asthma status and α-diversity measures (figure 2B–D), total bacterial load, or *H. influenzae* and *M. catarrhalis* abundance (figure 2E–G). However, prior to FDR, the relative abundance of *Streptococcus* and *Granulicatella* was found to be higher in participants with asthma, while five genera (*Campylobacter, Peptostreptococcus, Leptotrichia, Fusobacterium* and *Mogibacterium*) were less abundant (figure 2H, online supplemental figure 3). Post FDR, only *Granulicatella* was significantly higher in asthma.

Within-country analysis was then performed, with the exclusion of Brazil and Uganda due to low numbers of non-asthmatics. Microbiota composition differed between participants with asthma and without in Ecuador (R^2^=2.74%, p(perm)<0.001) and NZ (R^2^=1.98%, p(perm)=0.002), but not the UK (R^2^=1.49%, p(perm)=0.84). For Ecuador, this was characterised by lower α-diversity in participants with asthma (adjusted p<0.001), while there were no differences in α-diversity in NZ or the UK after FDR correction (figure 2I). Total bacterial load and abundance of *H. influenzae* or *M. catarrhalis* were not associated with asthma status in any country (figure 2I).

### Asthma inflammatory phenotypes are associated with sputum microbiota.

**Figure 3:**
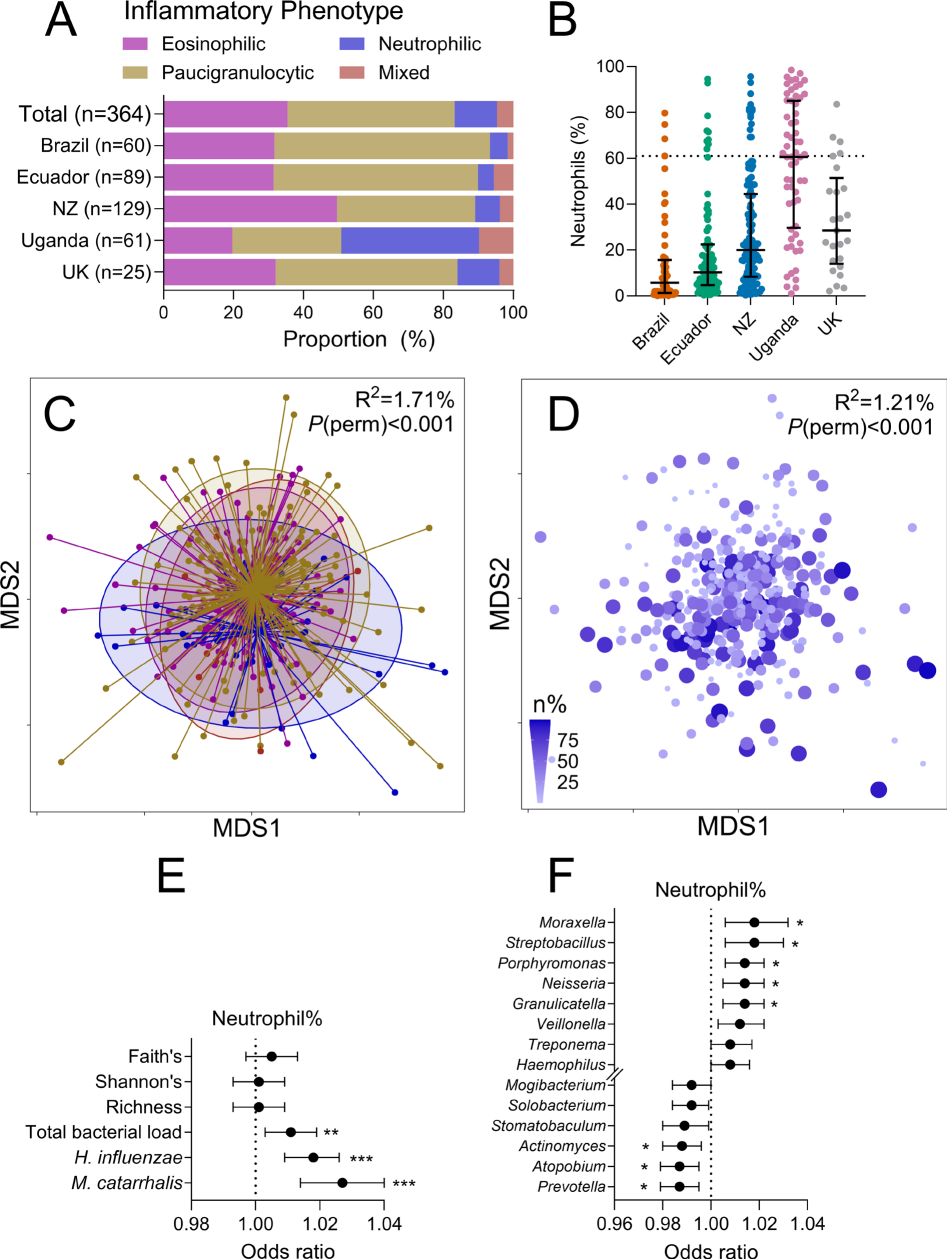
Sputum microbiota differs by inflammatory phenotype in asthma. (**A**) Distribution of inflammatory phenotypes by country. (**B**) Distribution of sputum neutrophil% by country. (**C**) Non-metric multidimensional scaling (NMDS) plot of Bray-Curtis dissimilarity by inflammatory phenotype where eosinophilic=purple, paucigranulocytic=gold, neutrophilic=blue, mixed=red. (**D**) NMDS plot showing dispersion by neutrophil percentage (n%). (**E**) Forest plot showing neutrophil% associated with α-diversity (Faith’s, Shannon’s, richness) and qPCR derived bacterial load (total, *Haemophilus influenzae* and *Moraxella catarrhalis*). (**F**) Forest plot showing taxa that differed significantly by neutrophil%. Statistics: (C) permutational multivariate analysis of variance including variables: inflammatory phenotype, country, age and sex; **(D**) Permutational multivariate analysis of variance including variables: neutrophil%, eosinophil%, country, age and sex; (**E, F**) Ordinal logistic regression including variables: neutrophil%, eosinophil%, country, age and sex; *** adjusted p<0.001, ** adjusted p<0.01, * adjusted p<0.05. qPCR, quantitative PCR.

The relationship between microbiota composition and inflammatory phenotypes (EA, NA, PGA and MGA; figure 3A,B) was assessed in participants with asthma. Microbiota composition differed significantly between phenotypes (R^2^=1.71%, p(perm)<0.001, figure 3C, online supplemental table 5). Pairwise analysis revealed these differences to be most pronounced between NA and PGA (pseudo-F=3.56, p(perm)<0.001) and between NA and EA (pseudo-F=3.50, p(perm)<0.001), with EA also significantly different to PGA (pseudo-F=2.25, p(perm)=0.015, online supplemental table 5). Total bacterial load and α-diversity did not differ between inflammatory phenotypes; however, *H. influenzae* was higher in NA compared with EA (adjusted OR (OR)=2.37 (95% CI 1.18 to 4.78); p*=*0.016; adjusted p=0.096) and PGA (aOR=3.31 (95% CI 1.67 to 6.54); p<0.001; adjusted p=0.0036, online supplemental figure 4). Analysis of differences in taxon distribution using Linear discriminant analysis Effect Size (LEfSe) identified an unassigned Lachnospiraceae taxon as enriched in PGA (LDA score=3.5; adjusted p=0.032), but no other significant associations.

When each inflammatory phenotype was compared with the non-asthmatic group, significant divergence was observed for NA (pseudo-F=3.59, p(perm)<0.001) and EA (pseudo-F=2.93, p(perm)=0.003), but not for PGA (pseudo-F=1.40, p(perm)=0.14) or MGA (pseudo-F=1.06, p(perm)=0.40) (online supplemental table 5).

To further explore associations between airway inflammation and sputum microbiota composition, analyses were performed using both neutrophil% and eosinophil% as separate continuous measures within the same model. Both neutrophil% and eosinophil% were significantly associated with microbiota composition, with this relationship being more pronounced for neutrophil% (neutrophil%: R^2^=1.21%, p(perm)<0.001, figure 3D; eosinophil%: R^2^=0.49%, p(perm)=0.033, online supplemental figure 5A; table 2).

**Table 2:** Permutational multivariate analysis of variance output assessing the independent effect of sputum neutrophil and eosinophil percentage in participants with asthma

	R^2^ (%)	Pseudo-F	p(perm)	Significance
Univariate				
Neutrophil %	1.21	4.43	<0.001	***
Eosinophil %	0.45	1.62	0.087	
Multivariate				
Neutrophil %	1.21	4.79	<0.001	***
Eosinophil %	0.49	1.94	0.033	*
Age	0.90	3.56	<0.001	***
Sex	0.46	1.80	0.051	
Country	7.65	7.58	<0.001	***

Significance codes: *** p<0.001, * p<0.05.

No α-diversity metric was associated with either neutrophil% (figure 3E) or eosinophil% (online supplemental figure 5C). However, neutrophil% (figure 3E), but not eosinophil% (online supplemental figure 5C), was associated with a higher bacterial load (adjusted p*=*0.0089). Prior to FDR correction, neutrophil% was positively associated with eight taxa and negatively associated with six (figure 3F). These included positive associations with relative abundance of *Haemophilus* and *Moraxella*, which was also reflected by positive association with absolute abundance of *H. influenzae* and *M. catarrhalis* (figure 3E). Following FDR, five taxa were positively associated and three negatively associated. Analysis based on eosinophil% identified positive associations with *Streptococcus*, *Streptobacillus* and *Actinobacillus*, and negative associations with *Prevotella* and *Leptotrichia* prior to FDR, with only *Streptococcus* significant after FDR correction (aOR 1.04 (95% CI 1.02 to 1.06); adjusted p=0.042; online supplemental figure 5D).

### Inflammation-microbiota relationships are country-specific

We next assessed whether the associations between markers of airway inflammation and sputum microbiota characteristics were country specific. Due to the smaller number of participants from each country, this analysis was performed using inflammatory cell percentage, rather than phenotype groups, with the UK subgroup excluded. While the analysis across the whole study showed that neutrophilia was positively associated with total bacterial load and microbiota similarity, associations within each country were more varied. In NZ, neutrophil% was significantly associated with microbiota similarity (R^2^=2.51%, p(perm)<0.001, online supplemental table 6). Similar relationships in Brazil and Uganda did not achieve statistical significance, and no association was observed for Ecuador (online supplemental table 6).

**Figure 4:**
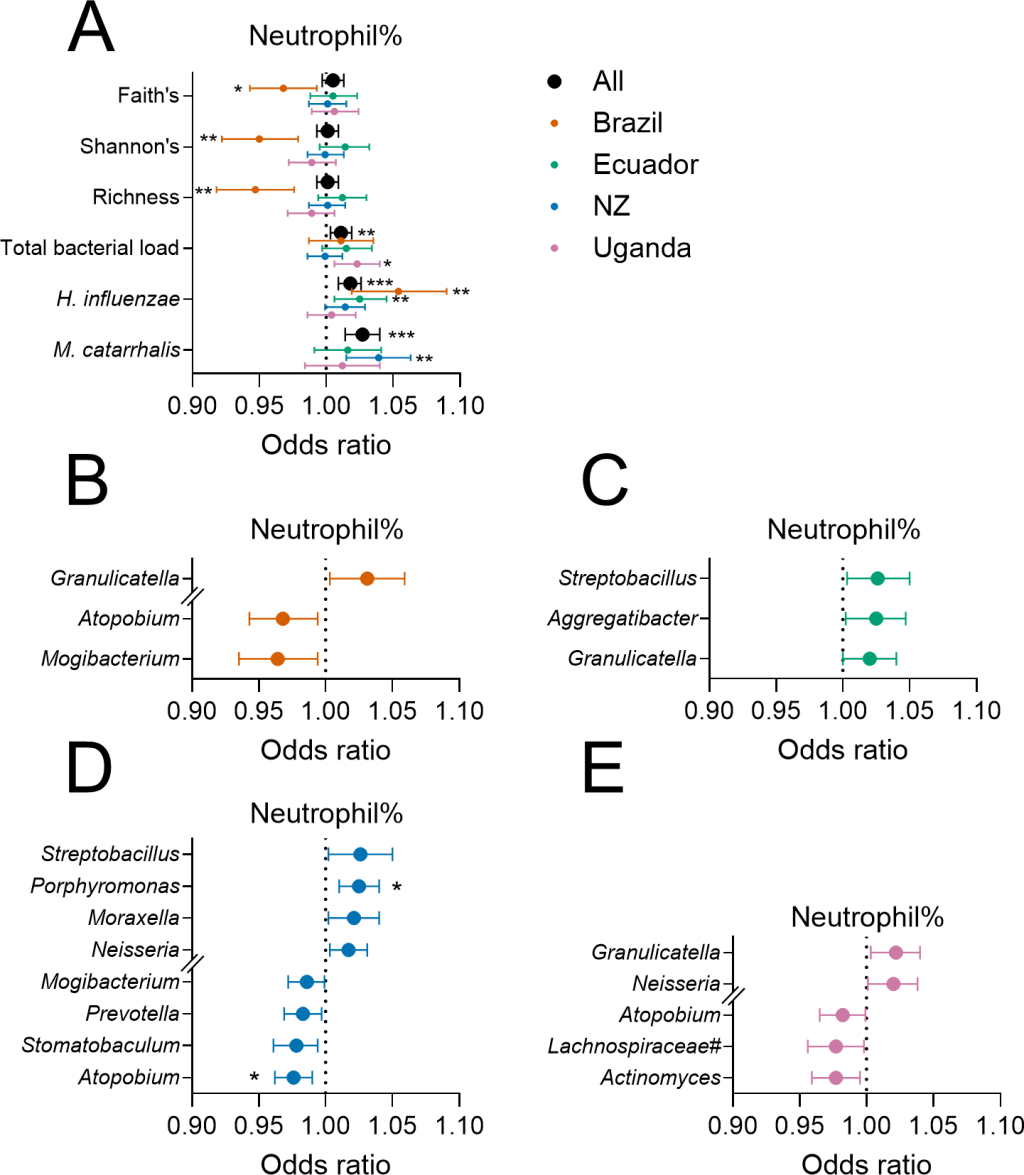
Within-country association between sputum microbiota and neutrophil%. **A**) Forest plot showing neutrophil% associated with α-diversity (Faith’s, Shannon’s, richness) and qPCR derived bacterial load (total, *Haemophilus influenzae* and *Moraxella catarrhalis*) in whole cohort (black), and within Brazil (orange), Ecuador (green), New Zealand (blue) and Uganda (pink). (**B**) Forest plot showing taxa that differed by neutrophil% in Brazil. (**C**) Forest plot showing taxa that differed by neutrophil% in Ecuador. (**D**) Forest plot showing taxa that differed by neutrophil% in New Zealand. (**E**) Forest plot showing taxa that differed by neutrophil % in Uganda. Statistics: ordinal logistic regression including variables: neutrophil%, eosinophil%, age and sex; *** adjusted p<0.001, ** adjusted p<0.01, * adjusted p<0.05. *M. catarrhalis* analysis was not performed for Brazil due to low detection frequency (three out of 60 participants). qPCR, quantitative PCR.

All three α-diversity metrics were inversely associated with neutrophil% in Brazil, but no other country (figure 4A**,** online supplemental figure 6B–D). Total bacterial load and abundance of *H. influenzae* and *M. catarrhalis* varied within countries, with total bacterial load positively associated with neutrophil% in Uganda, Brazil and Ecuador, but only reaching significance for Uganda (adjusted p*=*0.045, figure 4A, online supplemental figure 6A). *H. influenzae* and *M. catarrhalis* loads were similarly positively associated with neutrophil%, reaching statistical significance following FDR for *H. influenzae* in Brazil (adjusted p*=*0.0044) and Ecuador (adjusted p*=*0.048), and for *M. catarrhalis* in NZ (adjusted p*=*0.026) (figure 4A, online supplemental figure E,F).

Associations between neutrophil % and bacterial taxa relative abundance also differed between countries (figure 4B–E). Notably, prior to FDR, *Neisseria* was positively associated with neutrophil% in NZ and Uganda, while *Granulicatella* was positively associated with neutrophil% in Brazil, Ecuador and Uganda (figure 4B–E). Following FDR, only statistically significant associations remained for NZ, likely due to power.

Eosinophil% was not significantly associated with microbiota composition in any country, although a borderline statistically significant association was observed for Brazil (R^2^=3.08%, p(perm)=0.051, online supplemental table 6). Eosinophil% was inversely associated with Shannon’s diversity and richness in Uganda prior to FDR (but not statistically significant after FDR), and a trend towards a positive association was observed for NZ (online supplemental figure 7A). Total bacterial load and absolute abundance of *H. influenzae* and *M. catarrhalis* was not associated with eosinophil% in any country (online supplemental figure 6A). Of the eight taxa associated with eosinophil% prior to FDR (online supplemental figure 7B–E), *Streptococcus* was notable in being positively associated with eosinophil% in both Brazil and Uganda. However, no taxa remained significantly associated following FDR correction.

## Discussion

We previously reported that sputum inflammatory phenotypes in young people with asthma differed between the assessed countries, with higher levels of neutrophilic inflammation in Uganda and higher levels of eosinophilic inflammation in NZ.^9^ In this study, we extend our understanding of global heterogeneity of asthma by demonstrating the airway microbiota also reflects geographical and pathophysiological differences. Specifically, we report that airway microbiology differs geographically, with variation in microbiota characteristics across the five countries. In addition, we further support previous research that has described differences in airway microbiology between asthmatic and non-asthmatic participants^22^ and with varying levels of neutrophilic inflammation and with eosinophilic inflammation.^6 23–26^ In presenting our findings, we underscore the importance of international, comparative approaches to understanding asthma pathophysiology and the development of appropriate and effective treatment strategies.

We identified significant differences in airway microbiota characteristics between participant groups from each of the five countries. For example, the most pronounced differences in microbiota composition were observed between NZ and Uganda, while Brazil and Ecuador were most similar. Airway microbiota characterised in participants both with and without asthma from Uganda included high diversity and bacterial load, and a high abundance of *H. influenzae,* although with limited participants without asthma (n=8). While this represents the first prospective airway microbiota study spanning multiple diverse countries that is not confounded by technical limitations of meta-analyses,^27^ the high heterogeneity reported here aligns with meta-analyses in chronic obstructive pulmonary disease (COPD)^28^ and paediatric diseases.^29^ Together with more well-characterised gut microbiome studies,^30^ this body of research highlights region-specific microbial traits. The reasons likely reflect a range of factors including differences in early life development (eg, birth weight), environmental exposure (eg, air quality), lifestyle (eg, diet) and socioeconomic factors (eg, pathogen exposure) that may affect both the airway microbiota and development of specific asthma phenotypes. As we did not have individual-level information on these factors, we were not able to investigate this further.

Previous efforts to explore differences in the airway microbiota between asthma and non-asthma have focused largely on older populations in high-income settings.^3 25 31–35^ We identified a significant but modest difference in microbiota composition between asthmatic and non-asthmatic participants across the five countries assessed. While this was not associated with differences in diversity or bacterial load, several common oropharyngeal organisms were differentially associated with asthma, independent of age, sex and country. Associations between asthma and airway microbes in the literature vary, but there are some consistencies with the findings reported here. Namely, the higher levels of *Granulicatella* and *Streptococcus* we observed in participants with asthma are supported by previous investigations by Durack *et al*^3^ and Goleva *et al*,^34^ respectively. In a separate study by Durack *et al*, the authors also reported lower levels of *Leptotrichia* and *Peptostreptococcus* in participants with asthma compared with without, again consistent with our findings.^32^ As yet, the basis for these associations is unclear.

Despite between-country differences in both airway microbiota characteristics and inflammatory phenotypes, a consistent association between these two variables was observed after adjustment for age, sex and country. Overall, neutrophilic inflammation was associated with an altered microbiota composition, showing a positive relationship with total bacterial load, abundance of *H. influenzae*, *M. catarrhalis*, as well as other potentially pathogenic taxa, such as *Neisseria* and *Veillonella*.

The association between airway neutrophilia and pathogen or pathobiont abundance in asthma is supported by findings of studies in adults in HICs^4 5 36–39^ and in adult COPD^40^ and may reflect specific bidirectional processes. For example, both increased and altered neutrophil function in asthma can facilitate airway colonisation by opportunistic organisms, while presence of pathobionts may promote neutrophil recruitment.^41^ This process may be most pronounced in participants from Uganda, whose airway pathophysiology appears to more closely resemble those adult airway disease traits (eg, increased neutrophils). This centre-specific pattern may be due to the greater burden of environmental exposures including indoor smoke from coal-fire cooking practices, housing conditions or previous infections, contributing to this neutrophil-pathogen relationship. However, in contrast to adult studies, participants from Uganda had higher microbiota diversity and an inverse association between neutrophils and microbiota diversity. This potentially reflects the younger age of participants assessed here, where more established neutrophilic disease in adult asthma and associated therapies is likely to exert a greater selective pressure on microbiota composition compared with younger individuals with milder disease.

In contrast to neutrophil levels, the association with eosinophilic inflammation with airway microbiota characteristics was less pronounced. Specifically, while microbiota composition of EA was significantly different to PGA and no asthma, the microbiota association with neutrophil% was 2.46 times stronger compared with eosinophil% (1.21% compared with 0.49%). Further, eosinophil% was not associated with bacterial load or the detection of potential airway pathogens. However, eosinophil% was positively associated with *Streptococcus* abundance*,* the most prevalent and abundant taxa. These findings are in keeping with previous studies that have reported various strengths of association between eosinophilic inflammation and the airway microbiota,^2 3 31 38 39 42^ from none or minimal^2 38^ to moderate or strong.^3 31^

Despite reduced power, within-country analysis also identified region-specific associations. A greater divergence in microbiota composition between participants with or without asthma was observed in Ecuador, characterised by lower microbiota diversity in participants with asthma. Similarly, in those with asthma, microbiota diversity was inversely associated with neutrophil% in Brazil, while pathobiont abundance was positively associated with neutrophil% in Brazil, Ecuador and NZ. The lack of an association between neutrophil% and *H. influenzae* and *M. catarrhalis* levels in the Ugandan cohort is notable, given that individuals from Uganda exhibited higher neutrophil%, total bacteria and these specific pathobionts. While a positive association between neutrophil% and total bacteria in Uganda was observed, the lack of an association with *H. influenzae* and *M. catarrhalis* may reflect the contribution of other pathobiont species. Indeed, *Neisseria* was positively associated with neutrophils in Uganda (although non-significantly after FDR), which has pathogenic potential.^43^

Whether the region-specific associations identified here represent specific mechanistic processes is unclear. However, the combined influence of differences in inflammatory phenotype prevalence and environmental, lifestyle and socioeconomic factors could be significant. Regardless, these differences highlight the need for regional consideration when studying associations between asthma phenotypes and airway microbiota. These relationships may also have implications for asthma management strategies in different geographical regions. For example, the efficacy of maintenance azithromycin therapy has been shown to be predicted by abundance of *H. influenzae* in adults with persistent uncontrolled asthma.^7^ Further studies exploring the relationship between *H. influenzae* and azithromycin response in adolescents/young adults, combined with this data showing geographic variation in the distribution of this organism, would inform the efficacy of country-specific deployment of this therapy.

Important strengths of this study were the international representation of asthma and the detailed assessment of inflammatory phenotypes and airway microbiology. To our knowledge, it represents the most diverse single asthma airway microbiota study to date. With such a diverse cohort, careful protocol alignment for sample collection and processing was necessary. All sputum DNA extraction, sequencing and bioinformatic processing were performed at a single site, with careful inclusion of extraction and sequencing controls to assess and account for batch effects. Further, all analyses were adjusted for age and sex to account for between-group variance. We also undertook multiple approaches to explore the inflammation-microbiota relationships, including the conventional four group assessment, as well as including both neutrophil% and eosinophil% in a single model to enable mutual adjustment.

There are several important considerations when interpreting our findings. While the WASP-biome cohort was representative of the original WASP study, global variance of asthma phenotypes is broad.^44^ The geographical variance we report can represent a wide range of contributors including region-specific microbial exposures, population density, social determinants of health, air quality (including PM_2.5_ levels), and climate. While we performed additional adjustment for variables including BMI and asthma medications (on a subset of 316 participants where data were available) and found no effect on the associations reported (data not shown), we were not able to assess the effects of other factors, as individual-level data were not available. The specific reasons for these geographical differences therefore remain unclear, but do not likely involve differences in BMI and asthma medication use. Second, asthma cases were identified from responses to the ISAAC questionnaire, which assesses factors such as wheezing, whistling in the chest or asthma medication use. While it does not include objective tests (such as bronchodilator response or airway hyperreactivity testing), this validated questionnaire is particularly applicable in LMICs, where clinical testing is difficult and rates of physician-diagnosed asthma are low.^45^ Finally, 16S rRNA amplicon sequencing used to assess the microbiota has limited taxonomic resolution beyond genus-level and does not capture fungi or viruses, which may be associated with asthma heterogeneity.

In conclusion, we report variance in associations between airway microbiology and inflammatory phenotypes between countries, highlighting the need for further global studies of asthma pathophysiology, with potential implications for diagnosis and selection of therapies. We also report conserved associations between the airway microbiota and inflammatory phenotypes across HICs and LMICs, particularly in NA. With growing efforts to personalise asthma treatment and management strategies, there is now a clear opportunity to further examine how immune-microbiota interactions may guide such approaches.

## Supplementary material

10.1136/thorax-2025-222965 online supplemental file 1

10.1136/thorax-2025-222965 online supplemental file 2

## Data Availability

Data are available in a public, open access repository. All data relevant to the study are included in the article or uploaded as supplementary information.
